# Cortical morphometric similarity gradient in schizophrenia and its association with transcriptional profiles and clinical phenotype

**DOI:** 10.1017/S0033291725000479

**Published:** 2025-03-27

**Authors:** Yong Han, Xiujuan Wang, Shumin Cheng, Pengyue Yan, Yi Chen, Ning Kang, Zhilu Zhou, Xiaoge Guo, Yanli Lu, Qi Wang, Xue Li, Xi Su, Han Shi, Qing Liu, Wenqiang Li, Yongfeng Yang, Luxian Lv

**Affiliations:** 1 Department of Psychiatry, Henan Mental Hospital, the Second Affiliated Hospital of Xinxiang Medical University, Xinxiang, China; 2 Henan Key Lab of Biological Psychiatry, International Joint Research Laboratory for Psychiatry and Neuroscience of Henan, Xinxiang Medical University, Xinxiang, China; 3 Henan Collaborative Innovation Center of Prevention and Treatment of Mental Disorder, Xinxiang, China

**Keywords:** schizophrenia, cortical morphometric similarity, gradient, transcriptional profiles, clinical phenotype, neurobiological pathways

## Abstract

**Background:**

Recent studies have increasingly utilized gradient metrics to investigate the spatial transitions of brain organization, enabling the conversion of macroscale brain features into low-dimensional manifold representations. However, it remains unclear whether alterations exist in the cortical morphometric similarity (MS) network gradient in patients with schizophrenia (SCZ). This study aims to examine potential differences in the principal MS gradient between individuals with SCZ and healthy controls and to explore how these differences relate to transcriptional profiles and clinical phenomenology.

**Methods:**

MS network was constructed in this study, and its gradient of the network was computed in 203 patients with SCZ and 201 healthy controls, who shared the same demographics in terms of age and gender. To examine irregularities in the MS network gradient, between-group comparisons were carried out, and partial least squares regression analysis was used to study the relationships between the MS network gradient-based variations in SCZ, and gene expression patterns and clinical phenotype.

**Results:**

In contrast to healthy controls, the principal MS gradient of patients with SCZ was primarily significantly lower in sensorimotor areas, and higher in more areas. In addition, the aberrant gradient pattern was spatially linked with the genes enriched for neurobiologically significant pathways and preferential expression in various brain regions and cortical layers. Furthermore, there were strong positive connections between the principal MS gradient and the symptomatologic score in SCZ.

**Conclusions:**

These findings showed changes in the principal MS network gradient in SCZ and offered potential molecular explanations for the structural changes underpinning SCZ.

## Introduction

Schizophrenia (SCZ), a complex mental illness affecting approximately 1% of the global population (Kahn et al., 2015), is associated with profound personal disability and imposes substantial economic and public health burdens (Chong et al., 2016; Marder & Cannon, 2019). SCZ is believed to arise from intricate interactions between genetic and environmental risk factors that influence early brain development and shape biological responses to life experiences (Howes & Murray, 2014). However, the primary causes of SCZ remain poorly understood (Kahn et al., 2015).

Investigating structural brain abnormalities is essential for advancing our understanding of the etiology, progression, and treatment efficacy of SCZ in clinical practice (Amann et al., 2016; Bora et al., 2011; Haijma et al., 2012; Shepherd, Laurens, Matheson, Carr, & Green, 2012). Voxel-based morphometry (Ashburner & Friston, 2000) and surface-based morphometry (Dale, Fischl, & Sereno, 1999) are widely used techniques for assessing cortical morphology, primarily utilizing T1-weighted magnetic resonance imaging (MRI) data, for examining cortical features such as gray matter (GM) volume, cortical thickness (CT), and surface area (SA). These indices provide insights into cerebral cortical microstructure and are thought to reflect distinct genetic and cellular mechanisms (Winkler et al., 2010). Despite significant progress, the structural changes and underlying biological mechanisms contributing to SCZ are not yet fully understood, limiting advancements in diagnostic and therapeutic approaches.

Traditional univariate studies of brain structure primarily focus on isolated MRI features (Bethlehem et al., 2022). However, a single structural MRI scan contains vast amounts of information across numerous features or phenotypes (Fischl, 2012). Recently, morphometric similarity (MS) networks have emerged as a promising framework for integrating multiple structural MRI features into biologically relevant single-subject connectomes (W. Li et al., 2017; Seidlitz et al., 2018). MS network estimates the morphometric similarity between brain regions by calculating pairwise correlations of regional feature vectors, such as CT and T1w/T2w ratios. This method has demonstrated the potential of structural similarity networks to link macroscale MRI phenotypes with their neurobiological substrates (Li et al., 2021; Morgan et al., 2019; Seidlitz et al., 2020; Zhang et al., 2021).

Classical neuroanatomy suggests that cortical spatial organization is non-random, influenced by developmental mechanisms and evolutionary processes (Pandya, Seltzer, Petrides, & Cipolloni, 2015; Vogt & Vogt, 1919). Mesulam has described a gradient trajectory in the rhesus monkey brain, extending from sensory to cognitive functions at the macroscale structural level (Mesulam, 1998). Margulies et al. developed a method called diffusion embedding, which captures gradients in connectivity patterns across cortical regions (Coifman et al., 2005; Haak, Marquand, & Beckmann, 2018). Compared to linear dimensionality reduction techniques, such as principal component analysis, diffusion embedding more effectively projects local and long-range connections into a common space.

The MS network exhibits complex topology (Seidlitz, Vasa, et al., 2018), and gradient-based approaches provide a valuable perspective for linking low-dimensional representations of cortical organization to human cognition (Margulies et al., 2016). Liao et al. conducted the first study on MS gradients, identifying that the principal MS gradient is reliably anchored by motor and sensory cortices at its extremes. This gradient reflects fundamental properties of cortical organization, encompassing gene expression, cytoarchitecture, myeloarchitecture, and evolutionary expansion, thereby offering a hierarchical framework for cortical morphological markers (Yang et al., 2021). Beyond MS gradients, Yao et al. integrated multifaceted functional regional activity and network topology metrics to develop a functional similarity network (Meng et al., 2022). Their analysis demonstrated that the gradients embedded within these networks exhibit cortical layer-specific associations with gene expression and layer thickness. These findings further underscore the significant value of gradient-based similarity network studies in characterizing neuroimaging biomarkers.

Recent studies indicate that the principal gradient of the MS network strongly correlates with cortical organizational features in healthy individuals, anchored by sensory and motor cortices (Yang et al., 2021). In individuals with major depressive disorder (MDD), this gradient is spatially associated with gene expression, enriched for neurobiologically relevant pathways, and differentially expressed across cell types and cortical layers (Xue et al., 2023). However, it remains unclear how the MS gradient is disrupted in SCZ or how it relates to disease-associated gene expression and clinical phenotypes.

**Figure 1.**
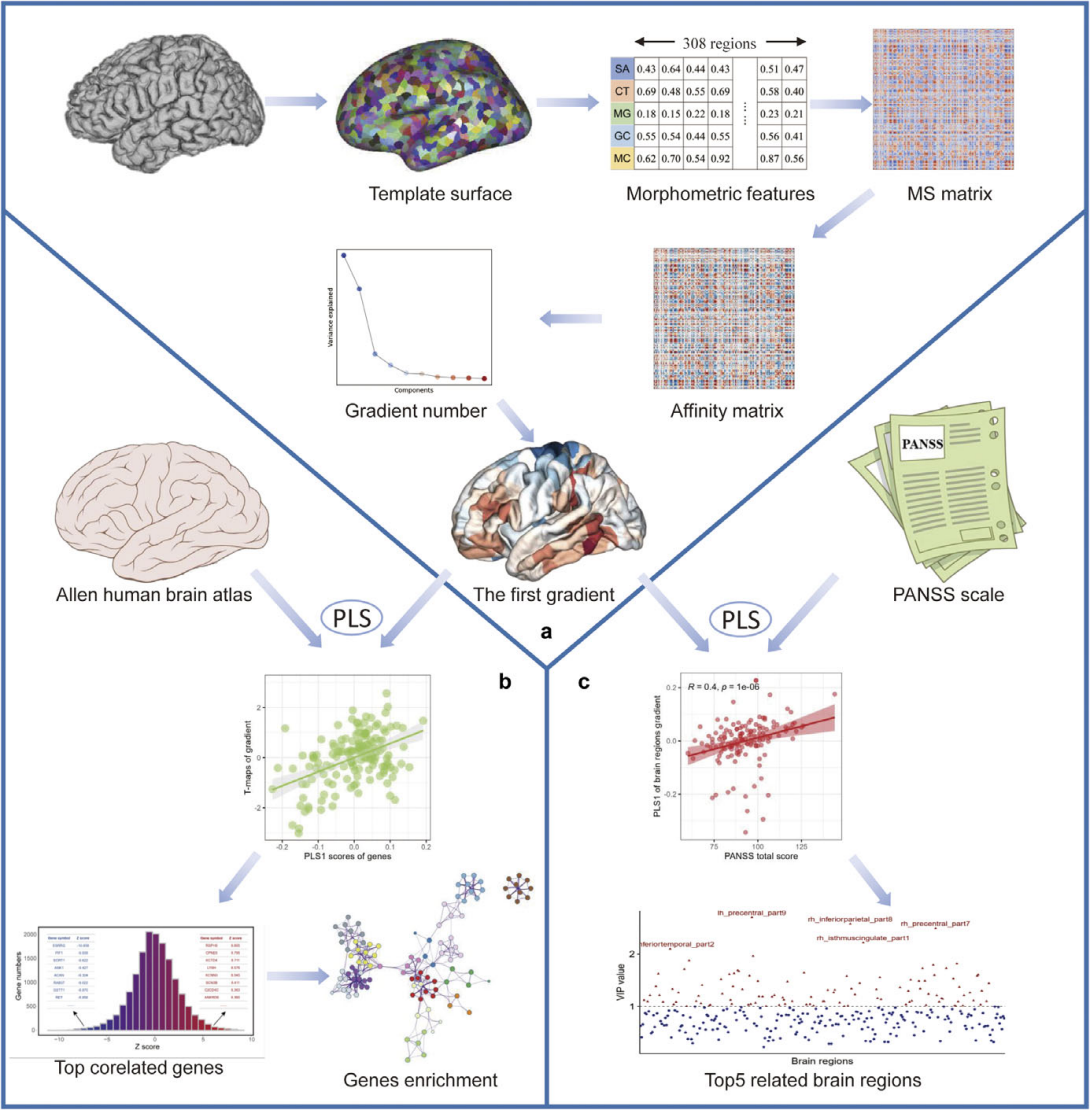
Diagrammatic summary of the study’s methodology. (a) Construction of gradients. The first step was to generate morphological features (GM, SA, CT, IC, and MC) from individual structural imaging maps. Regionally morphological features were extracted from the DK-308 atlas and combined into a vector in each region. The MS matrix was obtained from each individual, and Pearson’s correlation was determined between each pair of regional vectors. The affinity matrix was then created by applying a kernel function to the MS matrix. After using the diffusion embedding approach to deconstruct the affinity matrix, the first gradient map from each subject was obtained. (b) Transcriptional analysis. The Allen human brain atlas database was used to extract each gene’s expression value for each region of the left hemisphere, allowing for the creation of the gene expression matrix. PLS regression was used to correlate the principal MS gradient’s SCZ anomalies with the data on gene expression, and subsequent enrichment analyses on the significant gene list of first and second PLS components (PLS1 and PLS2) were carried out. (c) Clinical phenotype and brain regions analysis. The PANSS scale and its five factors scale were computed, and using PLS regression, the brain regions that had the strongest correlations with them were determined. CT, cortical thickness; DK, Desikan–Killiany; GM, gray matter; IC, intrinsic curvature; MC, mean curvature; SCZ, schizophrenia; MS, morphometric similarity; PANSS, Positive and Negative Syndrome Scale; PLS, partial least squares; SA, surface area.

This study aimed to investigate potential differences in the principal MS gradient between individuals with SCZ and healthy controls (HCs). Additionally, we sought to explore the relationships between these differences, gene expression, and clinical symptoms. Partial least squares (PLS) regression was employed to link SCZ-related alterations in the principal MS gradient to anatomically patterned gene expression, identifying candidate genes implicated in SCZ. Enrichment analyses further contextualized these findings by linking genes to molecular pathways, cell types, and cortical layers. Finally, correlations between changes in the principal MS gradient and clinical symptom variables were analyzed using PLS regression. A schematic overview of the methodology is provided in Figure 1.

## Methods

### Subjects

This study followed ethical guidelines and obtained approval from the ethical committee of the second affiliated hospital Xinxiang Medical University. The participants involved in the study provided written informed consent for the analysis of their data. Patients with SCZ were recruited from the inpatient department, and their diagnoses were determined by two trained psychiatrists using the diagnostic criteria of the structured clinical interview of DSM-IV (SCID). Healthy individuals, matched for age and gender, with no history of neurological or psychiatric disorders, were recruited from local communities. The general exclusion criteria encompassed age restrictions (younger than 18 years or older than 65 years), the absence of mental illness diagnosis according to the SCID-I nonpatient version, no history of psychiatric illness among first-degree relatives, and no instances of substance or alcohol dependence. Furthermore, on the same day as their MRI scans, all patients underwent scale assessments, including the Positive and Negative Syndrome Scale (PANSS) and the MATRICS Consensus Cognitive Battery (MCCB).

### MRI data acquisition

The MRI data in this study were obtained using 3.0T MR scanners from Siemens (Verio, Germany) and were acquired by a skilled radiological technician. A standard scanning head coil was used, and all participants were positioned in a supine position inside the MRI machine. During the scan, participants were asked to keep their eyes closed, remain awake, and stay still without engaging in any cognitive tasks. T1-weighted scans were performed using the following acquisition parameters: repetition time (TR) = 2,530 ms, echo time = 2.43 ms, inversion time = 1,100 ms, field of view (FOV) = 256 × 256 mm, matrix = 256 × 256, flip angle (FA) = 7°, layer thickness = 1 mm, isotropic voxel size = 1 mm^3^, no spacing, number of layers = 192, and scan time 480 s.

### Estimation of morphological parameters

We employed the FreeSurfer package 7.1.0 (http://surfer.nmr.mgh.harvard.edu) to conduct SBM analysis and obtain morphological measurements on the Ubuntu (version 12.04) platform. While detailed technical information regarding the use of FreeSurfer can be found elsewhere (Fischl, 2012), a succinct overview of the preprocessing procedure is provided. Specifically, non-brain structures, such as skulls, were removed, and images were registered to MNI305 space. The white surface, representing the interface between grey matter and white matter, as well as regions containing grey matter and cerebrospinal fluid/dura (known as the pial surface), were estimated within brain tissues. Following this, 3D cortical surface reconstruction was performed. Data quality control involved visual inspection and Euler value calculation (Rosen et al., 2018), resulting in consistent Euler values of 2 for both hemispheres across all participants, indicating reliable data. Using the DK-68 atlas (Romero-Garcia, Atienza, Clemmensen, & Cantero, 2012), which parcellated each region by employing a backtracking algorithm, cortical surfaces were then divided into 308 spatially contiguous regions (Vos de Wael et al., 2020; Siqi Yang et al., 2021). Inverse spherical normalization parameters, acquired during cortical surface reconstruction, were utilized to transform the parcellated DK anatomical template from standard space to each participant’s unique space. From these individual spaces, five structural features were extracted, including GM, CT, SA, intrinsic curvature (IC), and mean curvature (MC).

### Construction of MS gradients

We utilized the MS gradient derived from five features for the subsequent analyses, consistent with the methods established in previous studies conducted by the team of Liao, who introduced the MS gradient metric (Li et al., 2024; Yang et al., 2021). The morphometric features were z-score standardized over the 308 regions for every individual. Then, for each pair of z-score-adjusted morphometric feature vectors, Pearson’s correlation coefficients were computed. This produced a 308×308 matrix for each participant, known as the MS matrix. Preprocessing and MS gradients creation were done with the BrainSpace toolbox (Vos de Wael et al., 2020). In addition, we used two other features derived from T1 images, namely the curved index and folding index, to assess the robustness of the MS gradient. Li et al. and Yang et al. independently investigated the robustness of MS gradients to variations in the number of features by analyzing MS connectomes derived from seven metrics, which included five features based on the T1 image and two additional features derived from the diffusion-weighted image: fractional anisotropy and apparent diffusion coefficient (Li et al., 2024; Yang et al., 2021). Both studies observed that the spatial pattern of the MS gradient constructed from seven morphological features closely resembled the findings based on five features derived from T1w images. In another study, Li et al. employed a leave-one-feature-out approach to demonstrate the spatial similarities of regional MS gradients derived from the full set of seven features in T1w images and have confirmed the robustness of the regional MS gradients (Li et al., 2023).

### The MS gradient comparison

Examining between-group changes of the regional principal MS gradient while accounting for the effects of age, sex, age × sex, and education level was done using a general linear model (GLM). We compared the regional principal MS gradient alterations seen in the SCZ group to two well-established cortical area classifications: the Yeo atlas of the cortex, which is based on resting-state networks (Yeo et al., 2011), and the von Economo atlas of the cortex, which is based on cytoarchitectonic criteria (von Economo, Koskinas, & Triarhou, 2008). Using the GLM and regressing out the same covariates, we evaluated case-control differences in the principal MS gradient by computing the mean principal MS gradient score of all regions inside particular Yeo networks or von Economo classes. The significance of each region or network was corrected using the Benjamini–Hochberg false discovery rate (BH-FDR) method with a significance threshold of *p* < 0.05.

### Gene expression data preprocessing

The gene expression data, derived from six postmortem brains comprising a total of 3,702 distinct samples, was obtained from the AHBA database (Hawrylycz et al., 2012). We processed and mapped the transcriptome data onto the 308 parcellated brain regions using the abagen toolbox (https://www.github.com/netneurolab/abagen) (Markello et al., 2021). The preprocessing of the gene expression data involved several steps, which can be summarized as follows: (i) updating probe-to-gene annotations, (ii) applying an intensity-based filter, (iii) selecting probes, (iv) matching samples to brain regions, (v) handling missing data, (vi) normalizing samples, (vii) normalizing genes, (viii) calculating a sample-to-region combination metric, and (ix) selecting stable genes. Finally, a total of 15,633 genes remained. It is important to note that due to the inclusion of right hemisphere samples in only two out of the six brains from the AHBA database, analyses were limited to the left hemisphere (further details on the six donors can be found in Supplementary Table S1). Consequently, a gene expression matrix consisting of 152 regions by 15,633 genes was ultimately used for subsequent analyses.

### Transcription-neuroimaging association analysis

Utilizing PLS regression (Abdi, 2010), we investigated the connection between 15,633 genes’ expression and case-control differences in the major MS gradient, which is represented by *t* values from 152 left hemisphere cortical areas. In the PLS regression model, the independent variable was the *z*-score normalized gene expression matrix (152 regions × 15,633 genes), while the dependent variable was the *z*-score normalized principal MS gradient case-control *t* vector (152 regions × 1). The PLS components, consisting of linear combinations from weighted gene expression values, were ranked according to the explained variances between dependent and independent variables. Consequently, the top PLS component offered the most efficient low-dimensional representation for the covariance inherent in high-dimensional data matrices (Abdi & Williams, 2013).To determine the significance of the explained variance of the PLS components, we employed a spatial autocorrelation-preserving permutation test known as the spin test, with 10,000 permutations (Váša et al., 2018). Additionally, a permutation test method was used to assess the significance of genes contributing to the components. Only genes with statistical significance (*p* < 0.001) after BH-FDR correction were retained for further analyses.

### Enrichment analysis

Functional annotations of the genes were carried out using the Gene Ontology (GO) embedded in Metascape (https://metascape.org) (Zhou et al., 2019). To determine cortical layer enrichment, marker genes identified in a previous transcriptomic study were employed (He et al., 2017). Additionally, the cell-type specific expression analysis (CSEA) tool (http://genetics.wustl.edu/jdlab/csea-tool-2/) was used to perform developmental gene expression enrichment analysis to examine developmental time windows across brain regions (Dougherty, Schmidt, Nakajima, & Heintz, 2010). A significance criterion of *p* < 0.05 was applied to all enrichment analyses when adjusting them using the BH-FDR correction.

### Spatial spin null model

To mitigate the potential confounding effects of spatial autocorrelations, a spin test was conducted (Alexander-Bloch et al., 2018; Váša et al., 2018). This test involved randomly rotating the spherical projections of spatial maps while maintaining the spatial link. The purpose of this procedure was to generate a null distribution of Pearson’s correlation coefficients. To establish this null distribution, 10,000 spin permutation tests of the cortical areas were performed. These permutations were used to calculate the *p_spin_* value, which represents the proportion of null correlation coefficient values that exceeded the empirical correlation coefficient observed in the present study.

### Clinical phenotype-neuroimaging associations

The mean values from brain regions exhibiting the unusual principal MS gradient in the SCZ group were harnessed to undertake PLS analysis, consequently probing the link between this principal MS gradient and select clinical variables, i.e. the PANSS, amongst patients with SCZ. Aligning with the five-factor model (Wallwork, Fortgang, Hashimoto, Weinberger, & Dickinson, 2012), the PANSS scale was sectored into five distinct factors: depression, disorganization, excitement, negativity, and positivity. These factors were then employed as response variables, while the principal MS gradients across 308 brain regions were utilized as a predictive variable during the PLS analysis. To quantify the impact of individual brain regions on the response variables, we calculated Variable Importance in Projection (VIP) scores for all 308 regions. VIP scores serve as a metric of variable significance in multi-variate data interpretation, supplying insights into the level to which a variable contributes towards explaining the variation in the response variables (Chong & Jun, 2005). Brain regions were deemed significant predictors within the PLS regression model, provided that their corresponding VIP scores surpassed 1. Through scrutinizing the VIP scores for each brain region, we, in turn, could evaluate the relevance of specific regions in relation to every factor on the PANSS scale.

## Results

### Data samples

We eventually included 203 patients with SCZ and 201 healthy participants in our study after meeting the criteria for the structural MRI data. Supplementary Table S2 provides specifics on the sample characteristics of the patients and controls. Regarding age or sex, there were no significant differences between the two groups. However, a notable difference in educational level was observed (*χ* ^2^ = 47.065, *p* < 0.05), with patients exhibiting lower levels of education.

### Case-control differences in MS gradient

**Figure 2.**
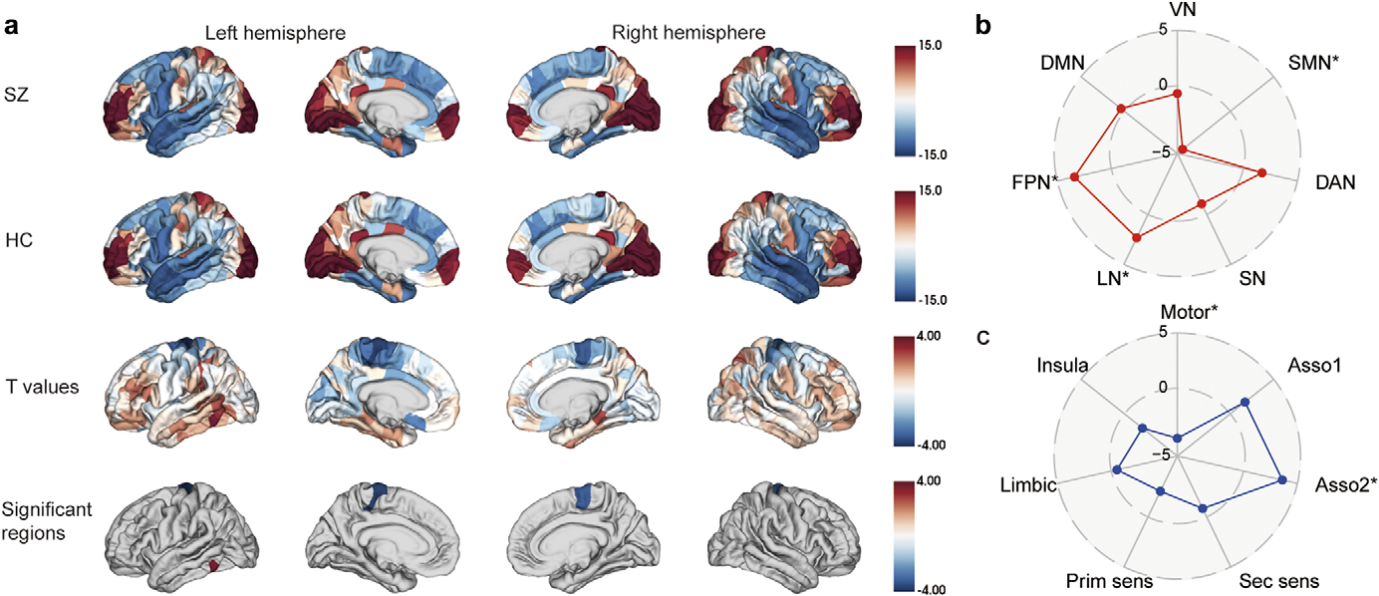
The principal MS gradient mapping in patients with SCZ and HCs. (a) The principal MS gradient pattern in patients with SCZ, HCs, *t*-value between them, as well as the statistically significant brain regions. (b) Functional community-based *t*-value (upper, Yeo functional networks) and cytoarchitecture-based *t*-value (lower, von Economo classes) of the principal MS gradient indicate significant differences primarily in the soma-tomotor network and primary motor class. Asso1, association cortex 1; Asso2, association cortex 2; DAN, dorsal attention network; DMN, default mode network; HCs, healthy controls; FPN, fronto-parietal network; Insula, insular cortex; Limbic, limbic regions; LN, limbic network; MS, morphometric similarity; Prim motor, primary motor cortex; Prim sens, primary sensory cortex; Sec sens, second sensory cortex; SMN, somato-motor network; SCZ, schizophrenia; VAN, ventral attention network; VN, visual network.

The first MS gradient scores, which explained 41% of the MS network variance in our dataset, were compared between patients with SCZ and healthy controls using the GLM by adjusted age, sex, age × sex, and education level as covariates. There was a significant case-control difference (*p* = 0.389) in the distribution of the mean principal MS gradient scores, as Figure 2a illustrates. Region-wise comparisons revealed that the principal MS gradient decreased in SCZ in the left hemisphere (lh) paracentral part 3, right hemisphere (rh) paracentral part3, rh precentral part 2, lh precentral part 3, and part 5, rh precentral part 2 regions, and an increase in the lh inferiortemporal part 4 region (Figure 2a and Supplementary Table S3). We then explored the alterations in the second and third gradients in SCZ, but no cortical regions showed statistically significant differences when compared to HCs (Supplementary Figure S1).

We employed two previous categorizations of cortical regions (Yeo’s 7-functional network atlas (Yeo et al., 2011) and the von Economo cytoarchitectural atlas (von Economo et al., 2008 to extend our findings to the functional and cytoarchitectural organization levels of the brain. With respect to the Yeo functional network atlas, SCZ patients displayed a diminished principal MS gradient in the somato-motor network, fronto-parietal network, and limbic network (FDR *p* < 0.05, Figure 2b and Supplementary Table S4). Concerning the von Economo cytoarchitectural atlas, SCZ patients demonstrated a reduced principal MS gradient in the motor cytoarchitectural class and association cortex2 class (FDR *p* < 0.05, Figure 2b and Supplementary Table S5).

To assess the stability of the regional principal MS gradient, we compared the spatial similarities between the regional MS gradients derived from the full set of seven features and those derived from a reduced set of five features. A strong correlation was observed between the two MS gradients (*r* = 0.985; *p* = 9.085e-236), as illustrated in Supplementary Figure S2.

### Transcription-neuroimaging associations

**Figure 3.**
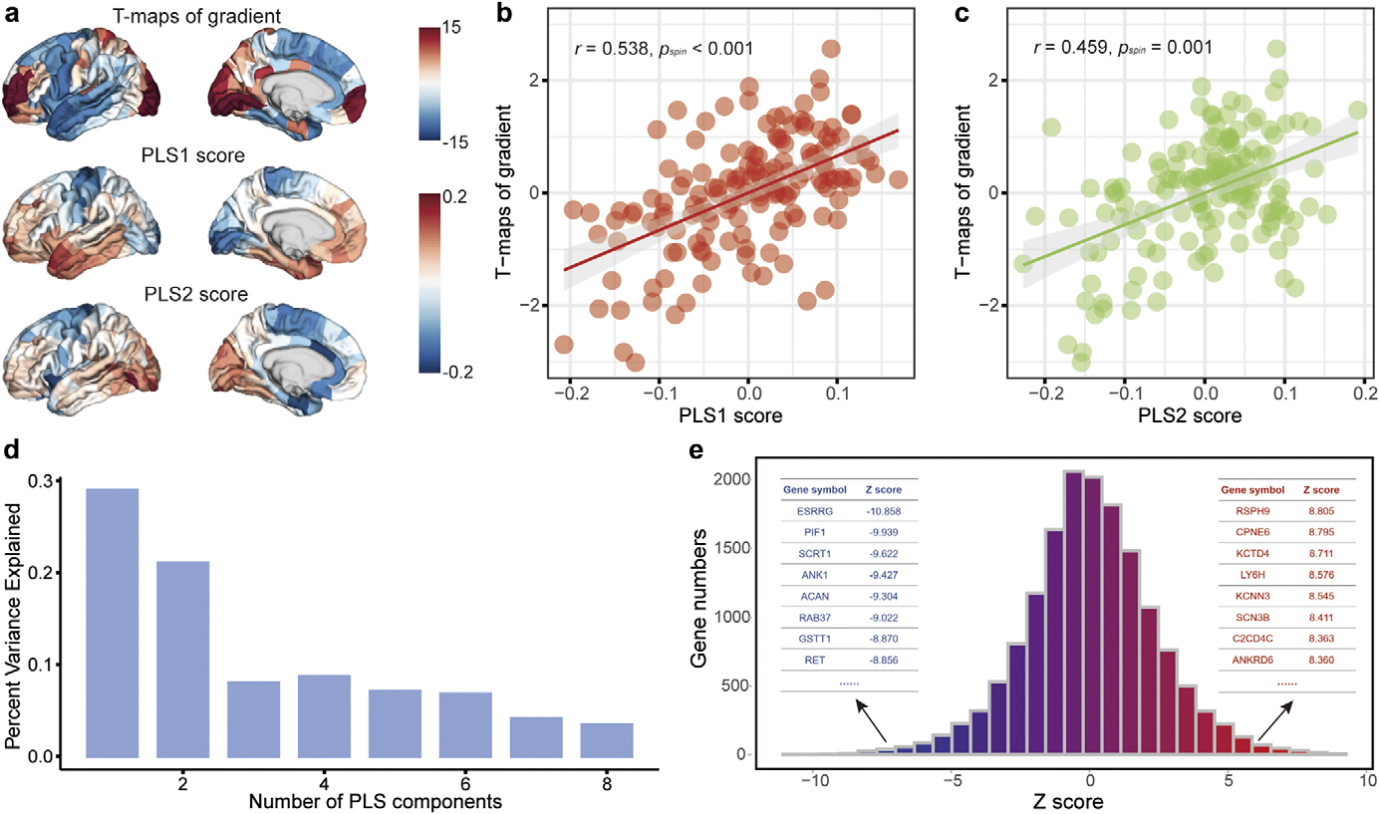
Gene expression profiles related to case-control differences of the principal MS gradient. (a) The case-control t-map of the regionally principal MS gradient scores in the left hemisphere, and the weighted gene expression map of regional PLS1 scores and PLS2 scores in the left hemisphere. (b, c) The scatterplot of the relationship of regional case-control changes in the principal MS gradient with regional PLS1 scores and PLS2 scores, respectively. The gray band indicates the 95% confidence interval. (d) The explanation of each PLS component for all genetic variations. (e) The *Z*-scores distribution of all genes and ranked PLS1 genes based on *Z* score. MS, morphometric similarity; PLS, partial least squares.

The brain gene expression matrix was procured from the AHBA database. Owing to the availability of only two right hemisphere data, we exclusively utilized the left hemisphere in this study. Consequently, the gene expression matrix (152 regions × 15,633 genes) was subjected to PLS regression to discern patterns of gene expression associated with the anatomical distribution of the principal MS gradient of case-control disparities (Figure 3a). The first PLS component (PLS1) accounted for 29.0% of the variance in the principal MS gradient case-control differences, considerably more than anticipated by chance (*p_spin_* = 0.015); the second PLS component (PLS2) explained 21.0% (*p_spin_* = 0.001) (Figure 3d). The distribution of PLS1 and PLS2 scores for gene expression is depicted in Figure 3e. Both the PLS1 gene expression map (*r* = 0.538, *p_spin_* < 0.001, Figure 3b) and PLS2 gene expression map (*r* = 0.459, *p_spin_* = 0.001, Figure 3c) exhibited a positive correlation with the case-control t-map of the MS gradient. In line with prior research (Li et al., 2021; Xue et al., 2023), we ranked the normalized weights of PLS1 and PLS2 according to each gene’s *z* score. In total, 2,446 genes were determined to make significant contributions to PLS1 and PLS2 (FDR *p* < 0.001, Figure 3e). The findings suggested that gene expression is associated with the principal MS gradient of differences between healthy controls and the SCZ group.

### Enrichment analysis of genes associated with the principal MS gradient

**Figure 4.**
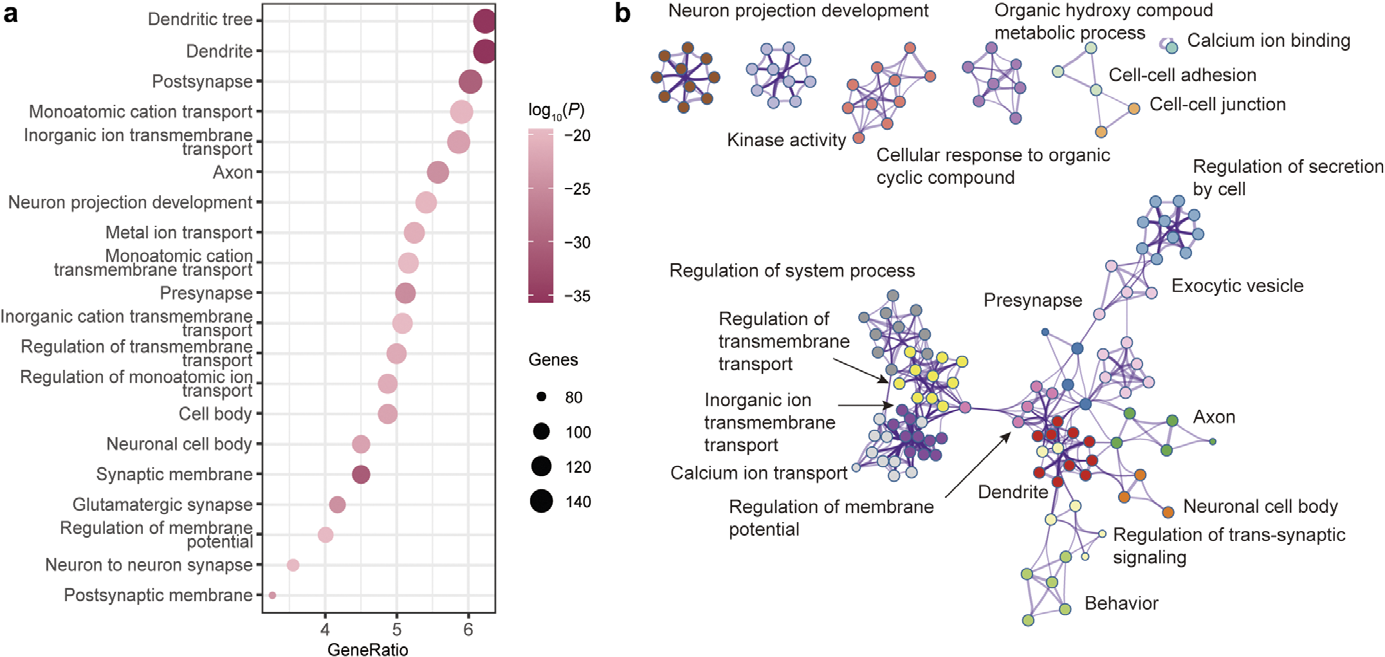
GO enrichment analysis of PLS genes. (a) The bubble plot shows the GO enrichment for the PLS genes. (b) Metascape enrichment network visualization showing the intra-cluster and inter-cluster similarities of enriched pathways. Each pathway is shown by a node, where the node size is proportional to the number of input genes included in the pathway, and different colors respond to different clusters.

**Figure 5.**
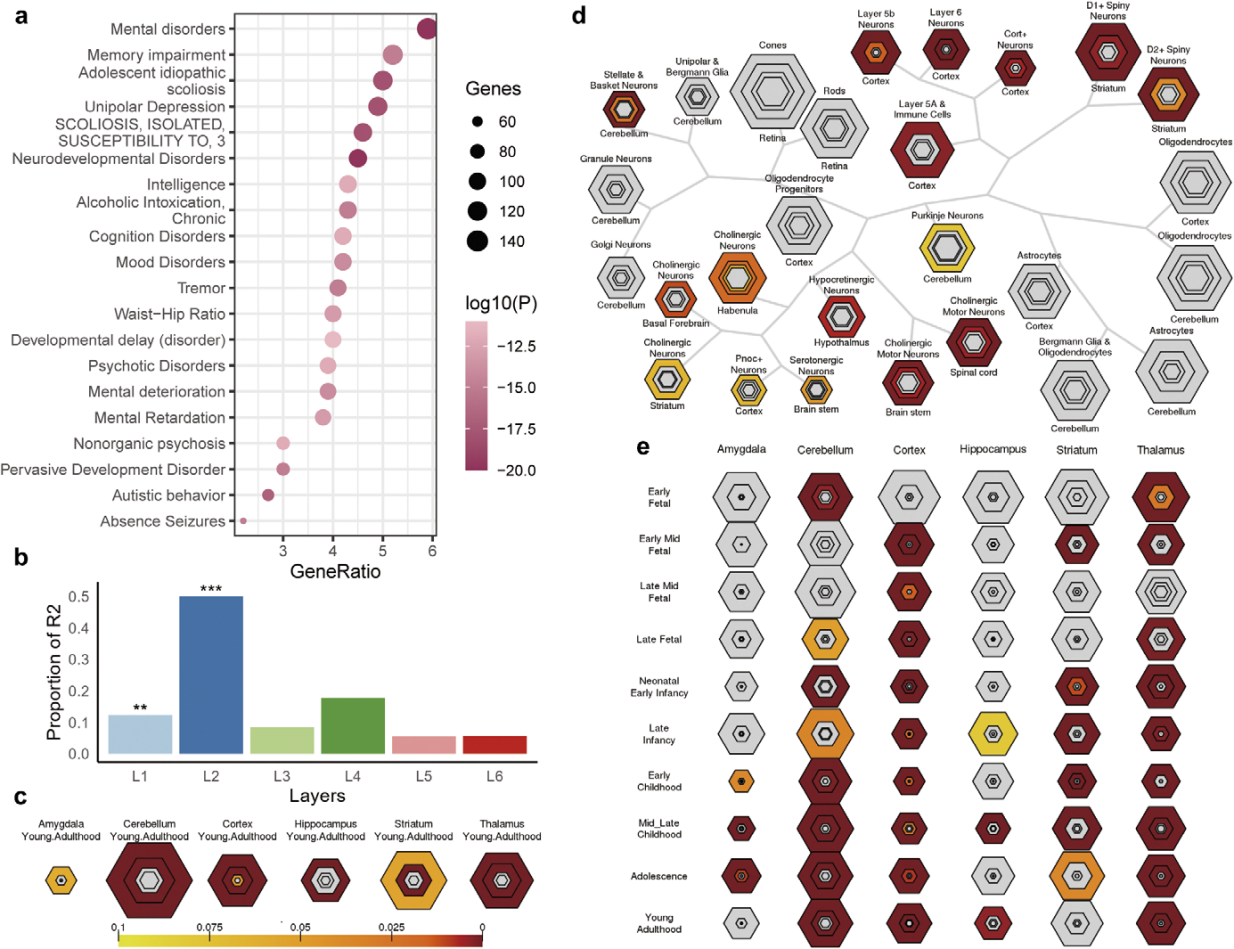
Disease enrichment analysis and SEA of PLS genes. (a) Disease enrichment analysis for the PLS genes. (b) Cortical layer enrichment analysis of the PLS gene list. * in (b) indicates that the statistical significance of the layer, ^**^ and ^***^ represent *p* < 0.05 and < 0.01, respectively. (c) Brain region SEA indicates that PLS genes are preferentially expressed in the cerebral cortex (corrected *q* = 0.03, pSI = 0.0001). (d) Cell type SEA indicates that PLS genes have higher expression levels in the neurons of several brain regions. (e) Development SEA indicates that the PLS genes show the most significant enrichment during young adulthood (corrected *q* = 2.541×10^−4^, pSI = 0.0001). The sizes of the bullseyes are scaled to the numbers of enriched genes at different thresholds (i.e. pSI = 0.05 [outermost], 0.01 [outer], 0.001 [inner], and 0.0001 [innermost]). The bullseyes are color‐coded according to the *q* values (BH‐FDR correction). SEA, specific expression analysis, PLS, partial least squares; GO, gene ontology.

Metascape was used for gene functional annotations, including enrichment of GO biological processes, GO cell components, GO molecular process, and disease and gene associations (disGeNET) analysis (Figures 4 and 5). These 2,446 genes are mainly enriched in synapse-related cellular components and neuron-related biological processes. Contrary to expectations, these genes did not exhibit enrichment for schizophrenia terms in the disease enrichment analysis. However, the enriched terms primarily consisted of psychiatric-related disorders. Utilizing the web server CSEA, we explored whether the genes were particularly enriched in specific human brain regions and developmental stages. The results divulged that the genes were expressed across a plethora of brain regions, including both the cortex and subcortex (e.g. thalamus, striatum, and amygdala).

### Correlations between the abnormal principal MS gradient and clinical variables

**Figure 6.**
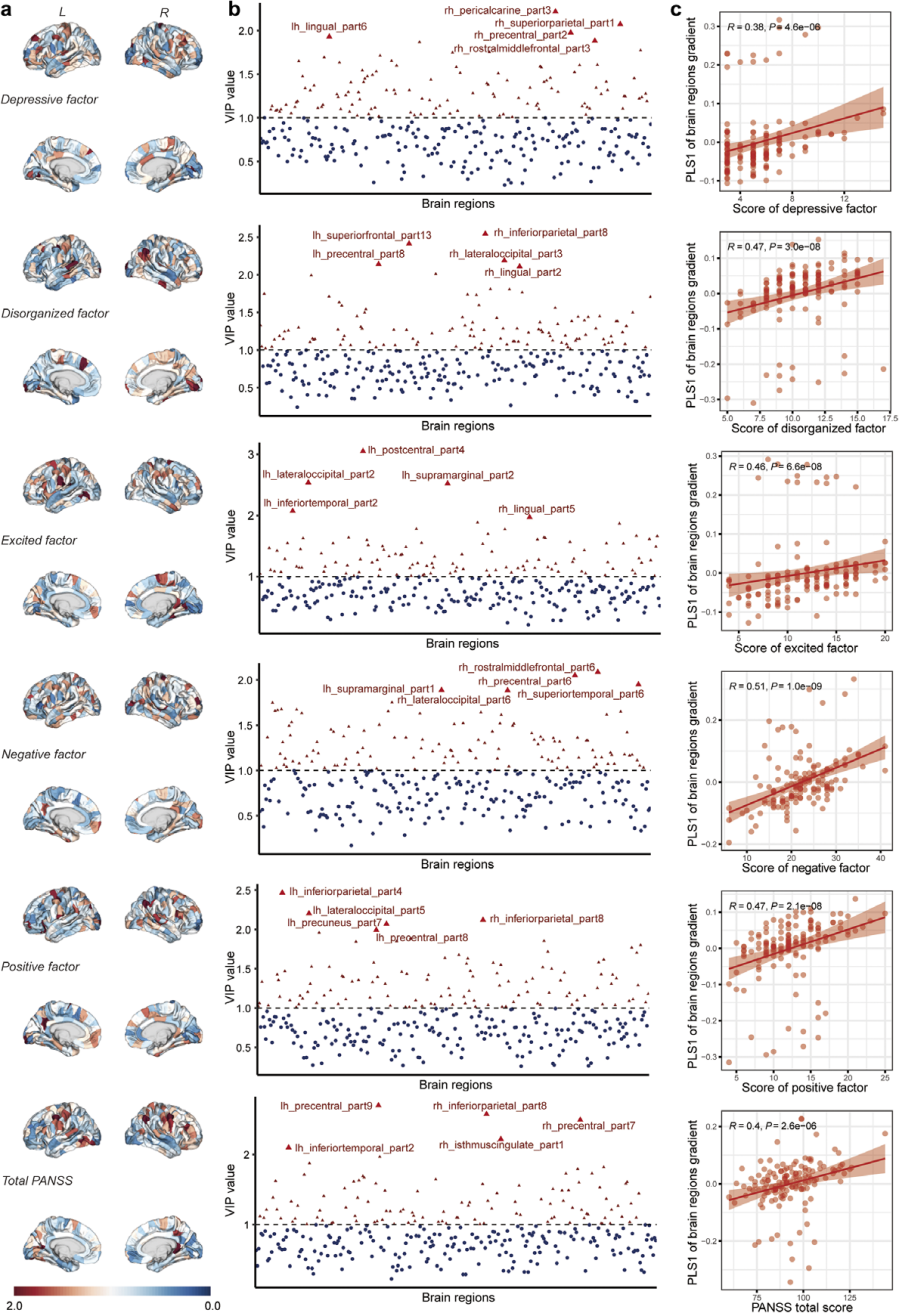
The VIP scores of brain regions correspond to the PANSS scale. (a) The brain mapping of VIP scores corresponding to five factors and total values of the PANSS scale. (b) The scatterplot of each brain region’s VIP scores corresponding to five factors and total values of the PANSS scale. The top 5 brain regions were highlighted and marked with the brain region name. (c) The scatterplot of PLS1 values of brain regions gradient with five factors and total values of PANSS scale. lh, left hemisphere; rh, right hemisphere; PANSS, positive and negative syndrome scale; PLS, partial least squares; VIP, variable importance in projection.

The correlations between the principal MS gradient scores of patients with SCZ and clinical variables of PANSS were assessed by using PLS regression. We use a five-factor model of PANSS as the response variable of Y and the principal MS gradient scores of each region as predictor variables of X. The result reveals a positive correlation between all five factors of PANSS and the first principal component of PLSX. This indicates that with the increasing principal MS gradient score, clinical symptoms further escalate (Supplementary Table S6). The VIP scores corresponding to the total score of PANSS and each individual factor across all brain regions are displayed in Figure 6. Among them, the brain region with the highest VIP score for the total PANSS score is lh precentral part9, while rh rostralmiddlefrontal part6 is identified as the brain region with the highest VIP score for the negative factor. For the positive factor, lh inferiorparietal part4 is associated with the highest VIP score, and for the excited factor, it is lh postcentral part4. Additionally, rh pericalcarine part3 exhibits the highest VIP score for the depressive factor, and rh inferiorparietal part8 is correlated with the highest VIP score for the disorganized factor.

## Discussion

In this study, we conducted the first investigation into the deviations of the principal MS gradient in both SCZ patients and healthy controls. Remarkably, we observed a spatial correlation between the principal MS gradient map of case-control differences and the cortical gene expression map. Furthermore, we identified that genes related to MS gradient changes were not only significantly enriched for neurobiologically consequential pathways but also exhibited preferential expression in diverse brain regions and cortical layers. This offers valuable insights into the relationship between the hierarchical organization of macroscopic morphometric profiles and microscopic transcriptomes during the progression of SCZ. Moreover, we delved into the brain areas associated with PANSS and subset factors.

The SCZ brain shows numerous neuroplasticity disruptions in combination with multiple variables (such as genetic, epigenetic, environmental, and neurodevelopmental) (Frost et al., 2004). We investigated the differences between individuals with SCZ and healthy controls using a sophisticated neuroimaging phenotype called the MS gradient. As a result of the complex topology of the morphometric similarity connectome (Yang et al., 2021), gradient approaches have instead used manifold-learning techniques to identify the main axes of variance in this connectome, providing a useful perspective for bridging low-dimensional representations of cortical organization and human cognition. MS gradient has the benefit of combining structural multi-features in a single individual when compared to structural covariance and anatomical connectivity (Seidlitz et al., 2020; Seidlitz, Vasa, et al., 2018; Wei, Scholtens, Turk, & Van Den Heuvel, 2018).

The principal MS gradient pattern is closely linked to fundamental cortical characteristics, including gene expression, cytoarchitecture, myeloarchitecture, and evolutionary expansion (Yang et al., 2021). Our principal MS gradient mapping found that both groups revealed that the prefrontal and parietal lobes have a larger MS gradient, while the temporal lobe has a lower MS gradient, and other brain regions tend to fall in the middle of the MS gradient. (Figure 2c). We discovered that changes in the principal MS gradient were mostly focused in areas associated with the somato-motor network (SMN) and primary motor network (Motor), such as the precentral and paracentral regions.

Although SCZ is traditionally defined by positive symptoms, and the disease’s negative symptoms are severe for many individuals, motor impairments are frequently present as well SCZ (Bernard, Goen, & Maldonado, 2017). Previous research revealed that SCZ is characterized by disrupted connectivity within the somatosensory-motor system and its links to subcortical and cortical executive networks, suggesting the interaction between brain functioning and behavioral features of SCZ (Kebets et al., 2019). Additionally, aberrations were also detected in the FPN, which has been implicated in numerous psychiatric and neurological conditions (Menon, 2011). Furthermore, the study revealed discrepancies between the LN in the Yeo functional network and the limbic class in the von Economo classes, which suggests the discordance between cytoarchitectonics-based and resting-state functional network-based parcellations of the brain.

In recent years, imaging genetics has surfaced as an interdisciplinary methodology that incorporates brain imaging techniques to assess the correlation between the human brain’s structural and functional phenotype, alongside the gene expression profile of the brain. This approach facilitates the comprehension of gene effects on behavior or psychiatric disorders, relying on more objective assessments of macrostructural evaluations (Fornito, Arnatkevičiūtė, & Fulcher, 2019; Morgan et al., 2019). SCZ arises from intricate interactions encompassing biological systems that span from genes and molecules to cells (Skene et al., 2018), networks (Dong, Wang, Chang, Luo, & Yao, 2018), and behaviors (Jauhar, Johnstone, & McKenna, 2022). This highlights the importance of leveraging in-depth analyses that consider the complexity of the disorder and its underlying influences to unveil its potential genetic roots and mechanisms.

Using the PLS approach, we observed a spatial correlation between the cortical map of case-control differences in the principal MS gradient and the cortical gene expression map. Furthermore, through the weighted combination of genes in the first and second PLS components, we identified potential drivers of structural hierarchical organization changes that mediate the genetic risk of SCZ. Our results demonstrated that the PLS gene list exhibited significant enrichment in cellular component terms such as dendrite, neuron, and axon, as well as in biological processes related to neuron projection development and regulation of trans-synaptic signaling. This suggests that neuronal impairment and maldevelopment may represent potential mechanisms of SCZ. Additionally, these genes were found to be predominantly enriched in psychiatric disorders, memory impairments, and neurodevelopmental disorders, indicating shared pathogenic genes or mechanisms among psychiatric disorders (Consortium et al., 2018; Cross-Disorder Group of the Psychiatric Genomics Consortium, 2013). This underscores the significance of the SCZ-related genes identified by PLS in offering novel insight into the complex substrates of SCZ.

Growing evidence suggests that understanding alterations in specific cell types within the central nervous system and human brain regions, as well as their contributions during specific developmental windows, can shed light on the pathophysiology of psychiatric disorders (Chana, Landau, Beasley, Everall, & Cotter, 2003; Nagy et al., 2020). In our study, we used cortical layer markers to investigate gene expression patterns in SCZ and found a significant enrichment of SCZ-related genes in cortical layers I and II, indicating that the distribution of different neuronal populations and their connections in these layers may play a crucial role in SCZ pathology (Chana et al., 2003). Additionally, Specific expression analysis (SEA) revealed that gene sets expressed in the cortex, cerebellum, thalamus, and hippocampus from early fetal stages to young adulthood were associated with SCZ. This implies that susceptibility to SCZ may occur during early life and extend into adolescence. These discoveries offer insightful information about prospective SCZ therapy targets.

Schizophrenia is characterized by a diverse range of symptoms encompassing alterations in perception, cognition, and emotion (American Psychiatric Association & Association, 2013). Exploring the relationship between symptom dimensions and brain changes is crucial for gaining insight into the underlying pathophysiological mechanisms of distinct psychiatric symptoms in schizophrenia, as well as providing precise localization of brain regions for neuromodulation interventions. The PANSS is a widely used questionnaire for evaluating symptomatology in schizophrenia, enabling investigation into dimension-specific abnormalities (Kay, Fiszbein, & Opler, 1987). In this study, we employed the PANSS five-factor model to examine the associations between each factor and specific brain regions. Through our analysis, we have identified the top five brain regions that exhibit the highest correlation with each PANSS factor. These brain regions could potentially represent pathological loci corresponding to the respective symptoms, offering valuable spatial information for further exploration of the etiology behind these symptoms.

There are several limitations to our study that need to be highlighted. To begin with, we created the MS gradient using only five morphometric characteristics. Future studies must still examine the hierarchical organization of morphology using multimodal imaging data, even though prior research has shown that T1-weighted restricted MS network construction is a suitable stand-in for multimodal MS networks when multimodal imaging is not possible (King & Wood, 2020; Seidlitz et al., 2020; Yang et al., 2021). Further research with bigger sample sizes is required to uncover anomalies in patients with SCZ, due to the relatively limited sample size of our study. Third, rather than using brain tissues from SCZ patients, our transcriptome analysis used AHBA, which is produced from healthy brain tissues. Therefore, the relationships between healthy transcriptomics and SCZ-dependent connection patterns examined here indicate components of the connectivity changes that affect large-scale organizations. Using gene expression data from a diseased cohort, which is not currently accessible, future investigations should verify our findings.

In summary, the results of this study confirmed our expectations regarding the altered principal MS gradient in individuals with SCZ and highlighted the spatial correlation with gene expression. Furthermore, we demonstrated that SCZ-related genes exhibited selective expression in various brain regions and cortical layers, and were enriched for neurobiologically significant pathways. Collectively, our findings provide novel insights into the disrupted coordination of structure in SCZ patients and may indicate a new endophenotype warranting further investigation into the complex substrate of SCZ.

## Supporting information

Han et al. supplementary material Han et al. supplementary material

## Data Availability

The Allen Human Brain Atlas database (http://human.brain-map.org/static/download) contains information on human brain gene expressions. The dataset (https://static-content.springer.com/esm/art%3A10.1038%2Fnn.4548/MediaObjects/41593_2017_BFnn4548_MOESM255_ESM.xlsx) contained several layer markers. The preprocessing software for neuroimaging is publicly accessible at http://surfer.nmr.mgh.harvard.edu/freesurfer/v7.1.0. The codes for both PLS and MS analysis are from https://github.com/SarahMorgan/Morphometric_Similarity_SZ. The programs for creating MS gradients are available at https://github.com/MICA-MNI/BrainSpace. Gene expression analysis codes are available at https://github.com/rmarkello/abagen. Enrichment analysis was carried out at https://metascape.org. The CSEA analysis was conducted using the following web app: http://genetics.wustl.edu/jdlab/csea-tool-2. The code for the spatial permutation test is available at https://github.com/frantisekvasa/rotate_parcellation.

## Supplementary material

The supplementary material for this article can be found at http://doi.org/10.1017/S0033291725000479.
