# Supplementary material for: Cortical morphometric similarity gradient in schizophrenia and its association with transcriptional profiles and clinical phenotype
Source: Psychol Med. 2025 Mar 27;55:e97. doi: 10.1017/S0033291725000479 (PMC12094636; doi:10.1017/S0033291725000479)
Supplement: Han et al. supplementary material [file S0033291725000479sup001.docx]

**Supplementary materials**

**Table S1.** The six donors’ information in the AHBA database.

**Table S1.** Demographic and clinical characteristics of schizophrenia patients and healthy control subjects.

**Table S3.** Regional differences between SCZ and healthy controls.

**Table S4.** The differences in the principal MS gradient in each Yeo network.

**Table S5.** The differences in the principal MS gradient in each von Economo class.

**Table S6.** The correlation of PANSS score with PLS1 values of brain regions gradient.

**Fig. S1.** The spatial patterns of the t-map for the first three morphometric similarity (MS) gradients comparing schizophrenia (SCZ) and healthy controls (HCs).

**Fig. S2.** Correlation between morphometric similarity (MS) gradients derived from seven and five morphometric features.

**Table S1.** The six donors’ information in the AHBA database.

| Donor ID | Sex | Age | Handedness | Hemispheres | Samples |
| --- | --- | --- | --- | --- | --- |
| H0351.2001 | Male | 24 | Left | Left + Right | 946 |
| H0351.2002 | Male | 39 | Left | Left + Right | 893 |
| H0351.1009 | Male | 57 | Cross-dominant | Left | 363 |
| H0351.1012 | Male | 31 | Right | Left | 529 |
| H0351.1015 | Female | 49 | Right | Left | 470 |
| H0351.1016 | Male | 55 | Right | Left | 501 |

**Table S2.** Demographic and clinical characteristics of schizophrenia patients and healthy control subjects.

| Characteristics | SCZ  (N =203) | HCs  (N =201) | *t*/*χ*^2^ | *p* value |
| --- | --- | --- | --- | --- |
| Age, mean (SD), year | 31.897±9.139 | 31.294±8.703 | 0.677 | 0.499 |
| Gender, male/female | 108/96 | 105/97 | 0.038 | 0.846 |
| Educational level |  |  | 47.065 | < 0.001 |
| (Unfinished) primary school | 20 | 7 |  |  |
| Middle school | 32 | 73 |  |  |
| High school | 46 | 53 |  |  |
| Junior college | 60 | 27 |  |  |
| University | 45 | 29 |  |  |
| Postgraduate | 11 | 1 |  |  |
| Duration of illness, mean±SD, year | 7.029±6.227 (n=203) ^*^ |  |  |  |
| PANSS scores, mean±SD |  |  |  |  |
| Total score | 92.683±13.825 (n=162) |  |  |  |
| Positive symptoms | 24.469±6.240 (n=162) |  |  |  |
| Negative symptoms | 25.080±5.999 (n=162) |  |  |  |
| General psychopathology symptoms | 43.025±8.558 (n=162) |  |  |  |

Note: ^*^ indicated that different n is due to missing values on one or more tests for some subjects. Abbreviations: PANSS, Positive and Negative Syndrome Scale; SD, standard deviation; SCZ, schizophrenia; HCs, healthy controls.

**Table S3.** Regional differences between SCZ and healthy controls.

| Regions | MNI coordinates  (x, y, z) | | | *t*-statistic | *p* value | FDR *p* value |
| --- | --- | --- | --- | --- | --- | --- |
| lh inferiortemporal part4 | 50 | -73 | -38 | 3.82 | 1.57×10^-4^ | 0.012 |
| lh paracentral part3 | -4 | -34 | 94 | -4.48 | 1.00×10^-5^ | 0.002 |
| lh precentral part3 | -52 | -19 | 82 | -4.01 | 7.10×10^-5^ | 0.007 |
| lh precentral part5 | -81 | -22 | 51 | -4.22 | 3.10×10^-5^ | 0.005 |
| rh paracentral part2 | -62 | -63 | -21 | -3.52 | 4.73×10^-4^ | 0.024 |
| rh precentral part2 | -1 | -63 | -67 | -3.72 | 2.30×10^-4^ | 0.014 |

Note: A GLM was used to investigate regionally principal MS gradient alterations in SCZ group, while regressing out the effect of age, sex, age × sex and education level. The *t*-statistic > 0 means healthy controls > SCZ, and the *t*-statistic < 0 means healthy controls < SCZ. FDR *p* value mean the *p* value after BH-FDR correction. Abbreviations: BH-FDR, Benjamini-Hochberg false discovery rate; GLM, general linear model; lh, left hemisphere; rh, right hemisphere; SCZ, schizophrenia.

**Table S4.** The differences in the principal MS gradient in each Yeo network.

| Statistics | VN | SMN | DAN | VAN | LN | FPN | DMN |
| --- | --- | --- | --- | --- | --- | --- | --- |
| *t*-statistic | -0.126 | -4.455 | 2.055 | -0.455 | 2.604 | 3.564 | 0.846 |
| *p* value | 0.900 | < 0.001 | 0.041 | 0.650 | 0.010 | < 0.001 | 0.398 |
| *FDR p value* | 0.900 | < 0.001 | 0.071 | 0.758 | 0.022 | 0.001 | 0.557 |

Note: A GLM was used to investigate regionally principal MS gradient alterations in SCZ group, while regressing out the effect of age, sex and age × sex. The *t*-statistic > 0 means healthy controls > SCZ, and the *t*-statistic < 0 means healthy controls < SCZ. FDR *p* value represent the *p* value after BH-FDR correction. Abbreviations: BH-FDR, Benjamini-Hochberg false discovery rate; DAN, dorsal attention network; DMN, default mode network; FPN, fronto-parietal network; GLM, general linear model; LN, limbic network; MS, morphometric similarity; SMN, somato-motor network; VAN, ventral attention network; VN, visual network.

**Table S5.** The differences in the principal MS gradient in each von Economo class.

| Statistics | Motor | Asso 1 | Asso 2 | Sec sens | Prim sens | Limbic | Insula |
| --- | --- | --- | --- | --- | --- | --- | --- |
| *t*-statistic | -3.560 | 2.029 | 3.746 | -0.265 | -1.825 | 0.027 | -1.372 |
| *p* value | < 0.001 | 0.043 | < 0.001 | 0.791 | 0.069 | 0.978 | 0.171 |
|  | 0.001 | 0.101 | 0.001 | 0.923 | 0.120 | 0.978 | 0.239 |

Note: A GLM was used to investigate regionally principal MS gradient alterations in SCZ group, while regressing out the effect of age, sex and age × sex. The *t*-statistic > 0 means healthy controls > SCZ, and the *t*-statistic < 0 means healthy controls < SCZ. FDR *p* value represent the *p* value after BH-FDR correction. Abbreviations: Asso1, association cortex1; Asso2, association cortex2; BH-FDR, Benjamini-Hochberg false discovery rate; GLM, general linear model; Limbic, limbic regions; Insula, insular cortex; Motor, primary motor; MS, morphometric similarity; Prim sens, primary sensory cortex; Sec sens, second sensory cortex.

**Table S6.** The correlation of PANSS score with PLS1 values of brain regions gradient.

| PANSS | *r* | *p* value | FDR *p* value |
| --- | --- | --- | --- |
| Depressive factor score | 0.3805 | < 0.001 | < 0.001 |
| Disorganized factor score | 0.4665 | < 0.001 | < 0.001 |
| Excited factor score | 0.4588 | < 0.001 | < 0.001 |
| Negative factor score | 0.5132 | < 0.001 | < 0.001 |
| Positive factor score | 0.4724 | < 0.001 | < 0.001 |
| Total PANSS score | 0.3970 | < 0.001 | < 0.001 |

Abbreviations: FDR, Benjamini-Hochberg false discovery rate; PANSS, positive and negative syndrome scale; PLS1, the first PLS component.


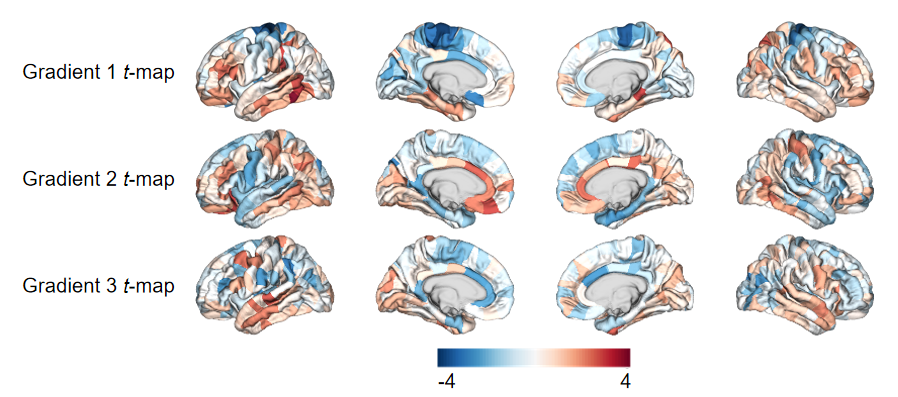


**Fig. S1.** The spatial patterns of the *t*-map for the first three morphometric similarity (MS) gradients comparing schizophrenia (SCZ) and healthy controls (HCs).


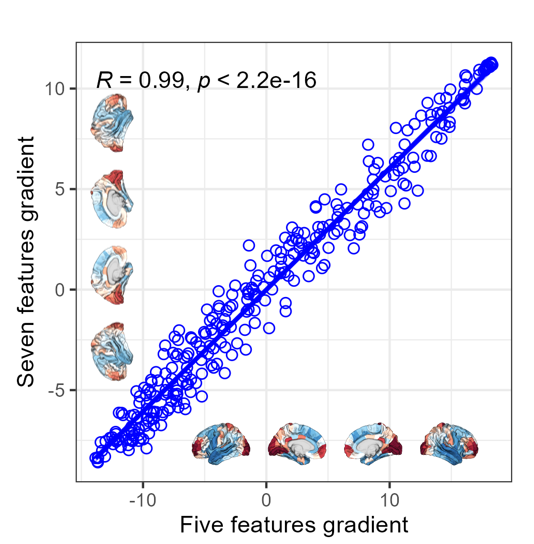


**Fig. S2.** Correlation between morphometric similarity (MS) gradients derived from seven and five morphometric features.
